# Powering care at the frontlines: healthcare providers' perspectives on solarised primary health centres in rural Karnataka, India, using the WHO HHFA framework

**DOI:** 10.3389/fmedt.2026.1800307

**Published:** 2026-05-07

**Authors:** Veeresh Tadahal, Rinshu Dwivedi, Ramesh Athe

**Affiliations:** 1 Department of Science and Humanities, Indian Institute of Information Technology, Tiruchirappalli, Tamil Nadu, India; 2 Department of Humanities and Social Sciences, National Institute of Technology, Hamirpur, Himachal Pradesh, India; 3 Data Science and Intelligence Systems, Indian Institute of Information Technology, Dharwad, Karnataka, India

**Keywords:** harmonized-health-facility-assessment, healthcare providers, primary healthcare, service readiness, solar energy, underserved region

## Abstract

**Introduction:**

Reliable electricity is fundamental to effective healthcare delivery; however, healthcare facilities in low- and middle-income countries continue to experience chronic energy shortage and frequent power interruptions. In rural India, Primary Health Centres (PHCs) often struggle to provide essential care due to unreliable electricity. This study explored healthcare providers’ perspective on the impact of solar energy interventions on healthcare infrastructure and service delivery in an aspirational district of Karnataka, India.

**Methods:**

A qualitative phenomenological study was conducted across the Yadgir district of North Karnataka, India. Focus Group Discussion (FGDs) and In-depth Interviews (IDIs) were carried out with 55 participants (16 men and 39 women), primarily healthcare providers, including medical officers, nurses, technicians, Accredited Social Health Activists (ASHAs), and other support staff. The interviews and discussions were audio-recorded, transcribed, translated into English, and thematically analysed. The study adhered to the Consolidated Criteria for Reporting Qualitative Research (COREQ) guidelines and applied the Harmonized Healthcare Assessment (HHFA) framework.

**Results:**

Four main themes emerged: (1) strengthened service availability; (2) enhanced service readiness; (3) advanced quality of care; and (4) improved data management, costs, operation, and maintenance. Solarisation of healthcare facilities significantly improved service quality, reliability, and resilience by enabling safe maternal deliveries, ensuring reliable vaccine cold storage, and supporting timely emergency care. The perspectives of healthcare providers offer a sustainable pathway to universal health coverage in underserved regions.

## 1 Introduction

Over the past decade, energy access priorities have expanded, moving beyond households and businesses to recognise the essential role of reliable energy in healthcare service delivery. Studies on health facility electrification increasingly emphasise the quality and reliability of electricity supply as critical determinants of healthcare outcomes, since many healthcare interventions depend directly on reliable power supply. Without electricity, the delivery of essential and critical care services becomes severely constrained (1). Healthcare facilities in many low- and middle-income countries (LMICs), particularly in sub-Saharan Africa and Asia, continue to face unreliable or absent electrical services, impacting patient and provider safety and healthcare service delivery (2). A study of 78 LMICs found that approximately 60% of healthcare facilities lacked reliable electricity, with frequent and prolonged power outages preventing the provision of safe, quality care (3). Empirical evidence from Ghana demonstrates a positive association between frequency of power outages and in-facility mortality, with mortality risk increasing by 43% for each day that electricity was unavailable for more than 2 h (4). Energy security influences key health system components, including workforce recruitment and retention. Energy access directly influences service availability by determining facility operating hours, nighttime service provisions, and emergency readiness (5). A review of nationally representative data from 11 sub-Saharan African (SSA) countries found that one in four health facilities lacked energy supply, while only 28% had reliable power supply (6). These deficits significantly affect facility functionality and compromise the equity, availability, and safety of essential healthcare services (7). Healthcare providers consistently identify non-functional medical equipment as a primary barrier to service delivery in facilities lacking reliable power supply (8). Power shortage is the single most common cause of medical equipment failure in LMICs, where up to 70% of devices are estimated to be broken or unused (9). The relationship between electricity access and workforce availability is particularly notable for female healthcare workers. Evidence from India indicates that PHCs with electricity—whether regular or intermittent—are significantly more likely to have a nurse on duty, whereas facilities without reliable electricity rarely report nursing availability (10).

Within the World Health Organization's (WHO) Harmonized Health Facility Assessment (HHFA) framework, power quality and reliability influence multiple dimensions of healthcare delivery. Service availability and readiness—defined as a facility's ability to meet patient health needs and deliver power-dependent services—are contingent on reliable power supply. Health facilities without reliable energy are unprepared to deliver adequate healthcare due to poor lighting, absence of refrigeration and sterilisation services, inefficient use of power-dependent medical equipment, and inability to attract trained staff. Quality of healthcare service provision is closely linked to power reliability, as stable power supply enables safe clinical procedures and adherence to infection prevention standards. For instance, WHO estimates indicate that infections resulting from unsterilised equipment affect approximately one in five postoperative patients in LMICs (9). Management and finance are significantly influenced by power reliability, as stable power supply supports staff satisfaction, effective information management systems, and overall facility operations. Health facilities with reliable power are better able to provide safe and comfortable working condition, contributing to improved staff retention, especially in the rural and remote areas. Despite increasing recognition of the importance of reliable electricity in healthcare delivery, limited research has examined the lived experiences and perspectives of healthcare providers regarding solar electrification of PHCs in rural India.

A comprehensive examination of rural healthcare facility electrification through solar energy necessitates emphasis on the lived experiences, challenges, and perspectives of healthcare providers, including medical officers, nurses, pharmacists, laboratory technicians, clerical staff, and other support staff serving the rural and remote areas. Their perspectives offer critical insights into how electricity access and its absence fundamentally shape healthcare delivery, patient outcomes, community trust, and health system resilience in underserved communities (11). The study focuses on healthcare provider perspectives on healthcare facility electrification through solar energy, using the WHO HHFA framework (12). It specifically examines PHCs located in rural areas of Yadgir district, Karnataka, India. Yadgir is situated in the northernmost part of Karnataka bordering Telangana and is classified as one of the state's most underdeveloped districts. It is included under the NITI (National Institute for Transforming India) Aayog Aspirational District Programme (ADP) and has the lowest Human Development Index (HDI-0.538) across education, health, nutrition, livelihood, and social outcomes (13). Evidence from Yadgir, one of the Aspirational Districts (ADs) of Karnataka, India assessed the impact of drought, also highlighted the concerns in terms of the health and other nutritional indicators lagging in these areas, where reliable energy can be cited as one of the contributing factors (14). This unreliability poses significant challenges for healthcare facilities, particularly at the PHC level, constraining the delivery of maternal and child healthcare, immunisation, emergency care, and other medical emergency services. In this context, the study aims to explore the perspectives of healthcare providers on the implementation and impact of solarisation of rural PHCs, with a focus on how power supply access and reliability affect healthcare service delivery, staff performance, and overall health system functioning. Despite the potential benefit of solar energy in healthcare facilities, there are limitations with regard to high upfront installation costs and post-implementation challenges related to system maintenance technical troubleshooting, affecting long-term sustainability.

## 2 Methods

This study followed the COREQ (Consolidated Criteria for Reporting Qualitative Research) guidelines. The rationale of the study was to understand the impact of solarisation on the PHCs from a healthcare provider's perspective. The selection of the participants was based on their consent and willingness to share experiences. A random sampling procedure was employed, resulting in a total of 55 participants (16 men– and 39 women) present at the PHCs during the field visits (Table 1). Data were collected through focus group discussions (FGDs) and in-depth interviews (IDIs), which were audio-recorded and transcribed with consent. A total of 10 FGDs (*n* = 55) were conducted in the blocks of Yadgir, Shahpur, and Surpur. FGDs were open-ended questions to explore participants’ experiences with PHCs before and after solarisation.

**Table 1:** Details healthcare provider respondents with gender, cadre, and age.

S. no.	District	Block	PHC and village name	Gender	Cadre	Age
1	Yadgir	Yadgir	PHC Ajalapur	Female	Staff Nurse	48
2	Yadgir	Yadgir	PHC Ajalapur	Female	ASHA	34
3	Yadgir	Yadgir	PHC Ajalapur	Male	HIO	54
4	Yadgir	Yadgir	PHC Balichakra	Female	Staff Nurse	38
5	Yadgir	Yadgir	PHC Balichakra	Male	Group D	28
6	Yadgir	Yadgir	PHC Balichakra	Female	Staff Nurse	29
7	Yadgir	Yadgir	PHC Balichakra	Female	Group D	32
8	Yadgir	Yadgir	PHC Balichakra	Female	ASHA	35
9	Yadgir	Yadgir	PHC Balichakra	Female	ASHA	38
10	Yadgir	Yadgir	PHC Balichakra	Female	ASHA	42
11	Yadgir	Yadgir	PHC Balichakra	Female	ASHA	35
12	Yadgir	Yadgir	PHC Elheri	Female	Staff Nurse	32
13	Yadgir	Yadgir	PHC Elheri	Female	Staff Nurse	34
14	Yadgir	Yadgir	PHC Elheri	Female	Group D	38
15	Yadgir	Yadgir	PHC Elheri	Male	Medical Officer	41
16	Yadgir	Yadgir	PHC Gajarakot	Female	Staff Nurse	36
17	Yadgir	Yadgir	PHC Gajarakot	Female	Trained Ayah	62
18	Yadgir	Yadgir	PHC Gajarakot	Male	Group D	29
19	Yadgir	Yadgir	PHC Gajarakot	Female	Lab Technician	31
20	Yadgir	Yadgir	PHC Kadechur	Female	Medical Officer	35
21	Yadgir	Yadgir	PHC Kadechur	Female	Staff Nurse	30
22	Yadgir	Yadgir	PHC Kadechur	Male	Group D	37
23	Yadgir	Yadgir	PHC Kadechur	Male	Lab Technician	39
24	Yadgir	Yadgir	PHC Mahadwar	Female	Staff Nurse	35
25	Yadgir	Yadgir	PHC Kotegera	Female	Staff Nurse	45
26	Yadgir	Yadgir	PHC Kotegera	Female	Group D	35
27	Yadgir	Yadgir	PHC Kandkur	Female	Staff Nurse	38
28	Yadgir	Yadgir	PHC Kandkur	Female	Trained Ayah	66
29	Yadgir	Shahpur	PHC Chamnal	Male	Group D	43
30	Yadgir	Shahpur	PHC Chamnal	Female	Staff Nurse	29
31	Yadgir	Shahpur	PHC Malar	Male	Medical Officer	45
32	Yadgir	Shahpur	PHC Malar	Male	Group D	38
33	Yadgir	Shahpur	PHC Malar	Female	Staff Nurse	35
34	Yadgir	Shahpur	PHC Malar	Female	Lab Technician	32
35	Yadgir	Shahpur	PHC Hattigudur	Male	Group D	38
36	Yadgir	Shahpur	PHC Hattigudur	Female	Staff Nurse	35
37	Yadgir	Shahpur	PHC Hattigudur	Female	Staff Nurse	32
38	Yadgir	Shahpur	PHC Hayyal B	Male	Group D	36
39	Yadgir	Shahpur	PHC Hayyal B	Female	Staff Nurse	32
40	Yadgir	Shahpur	PHC Tadibidi	Female	Staff Nurse	33
41	Yadgir	Shahpur	PHC Tadibidi	Male	Group D	51
42	Yadgir	Shahpur	PHC Tadibidi	Male	Group D	30
43	Yadgir	Shahpur	PHC Kurkunda	Female	Staff Nurse	47
44	Yadgir	Shahpur	PHC Kurkunda	Female	Group D	33
45	Yadgir	Shahpur	PHC Wandurga	Female	Medical Officer	35
46	Yadgir	Shahpur	PHC Wandurga	Female	Staff Nurse	32
47	Yadgir	Shahpur	PHC Wandurga	Female	Lab Technician	35
48	Yadgir	Shahpur	PHC Wandurga	Female	Group D	42
49	Yadgir	Shahpur	PHC Wandurga	Female	Staff Nurse	30
50	Yadgir	Shahpur	PHC Chatnahalli	Female	Staff Nurse	34
51	Yadgir	Shahpur	PHC Gogi	Female	Staff Nurse	43
52	Yadgir	Shahpur	PHC Gogi	Male	Group D	35
53	Yadgir	Shorpur	PHC Yemnur	Male	Group D	26
54	Yadgir	Shorpur	PHC Yemnur	Female	Staff Nurse	37
55	Yadgir	Shorpur	PHC Guttibasaweshwar	Male	Group D	35

The medium of communication was Kannada. Through data insights, thematic analysis was carried using MAXQDA software. Codes were generated based on the emerging patterns in the data and organised into main themes and subthemes based on the participants’ experiences and perspectives. Three major themes were adopted based on the theoretical framework of the WHO HHFA Framework. The FGD method allows community members to share their experiences and lived realities collectively regarding the impact of solarisation on healthcare outcomes. Data saturation was reached when no new information or dimensions emerged from successive FGDs, and additional FGDs yielded repetitive information, justifying the final sample size for the qualitative study. Qualitative data were collected through FGDs and interviews with sessions lasting approximately 55 - 60 minutes. All discussion were audio recorded in Kannda with informed consent and later transcribed for analysis.

### 2.1 Data management and analysis

Thematic analysis was applied to the transcriptions using a manual coding process to ensure accuracy and depth in identifying themes and categories. All the interviews were conducted in the Kannada language, voice-recorded, transcribed, and translated into English. Based on field notes and FGDs, a content analysis method was followed for data retrieval and interpretation of the underlying meaning of the data captured during the field visits. The perspectives shared by community members were further analysed and categorsied into themes and sub-themes, capturing lived experiences before and after solarization and the observed changes in healthcare services and outcomes, particularly in maternal, child and emergency healthcare services. Thematic analysis was employed, with two researchers coding the data independently to enhance reliability.

### 2.2 Ethical consideration

Ethical approval was obtained from the District Health Office, Yadgir, and the Ethical Review Board of SELCO Foundation, and reviewed by the academic supervisor at NIT Hamirpur. Written informed consent was obtained from all participants and their legal guardians. Measures were taken to ensure confidentiality, voluntary participation, and data anonymisation.

### 2.3 Conceptual framework

The HHFA was developed by the WHO in 2016 as a part of its broader effort to strengthen health information systems and monitor progress towards universal health coverage (UHC). The HHFA framework was officially released in 2018 as a standard approach for countries to assess service availability, service readiness, quality of care, and management and governance. It consolidates earlier methodologies, including WHO’s Service Availability and Readiness Assessment (SARA, 2012) and United Nations Agency for International Development's (USAID) Service Provision Assessment (SPA, 1980), into a single harmonised tool. The HHFA is a comprehensive, globally recognised gold-standard framework. It has a strong emphasis on primary healthcare, basic hospital services, and the capacity to deliver services and a well-functioning health facility essential for achieving UHC. The HHFA framework comprises the following four key domains:

1. Service availability parameters assess the physical presence of essential healthcare services and resources within the facility. This includes essential infrastructure like buildings, beds, staff, basic equipment, diagnostics, and specific health services offered. Indicators reflect a healthcare facility’s capacity to provide a range of services reliably to the community. The major indicators are range and type of services, infrastructure, and staff availability.
2. Service readiness estimates whether the prerequisites for providing quality healthcare services are present and functional. It focuses on the functionality of essential equipment, trained healthcare workers, medicines, guidelines, and infection prevention control systems. Readiness reflects capacity to deliver the services that should be available, ensuring all components work together effectively.
3. Quality of care focuses on the healthcare services provided in accordance with established clinical protocols and standards. It assesses the provider competency, adherence to patient care processes, patient safety measures, and overall effectiveness of care. It reflects the process and outcome dimensions of service provision.
4. Management and finance parameters assess whether the facility has appropriate governance, financial management, and administrative systems to support continuous availability and quality of healthcare services. It includes evaluation of facility management, staff supervision, quality assurance systems, record-keeping, and health information systems.

**Figure 1:**
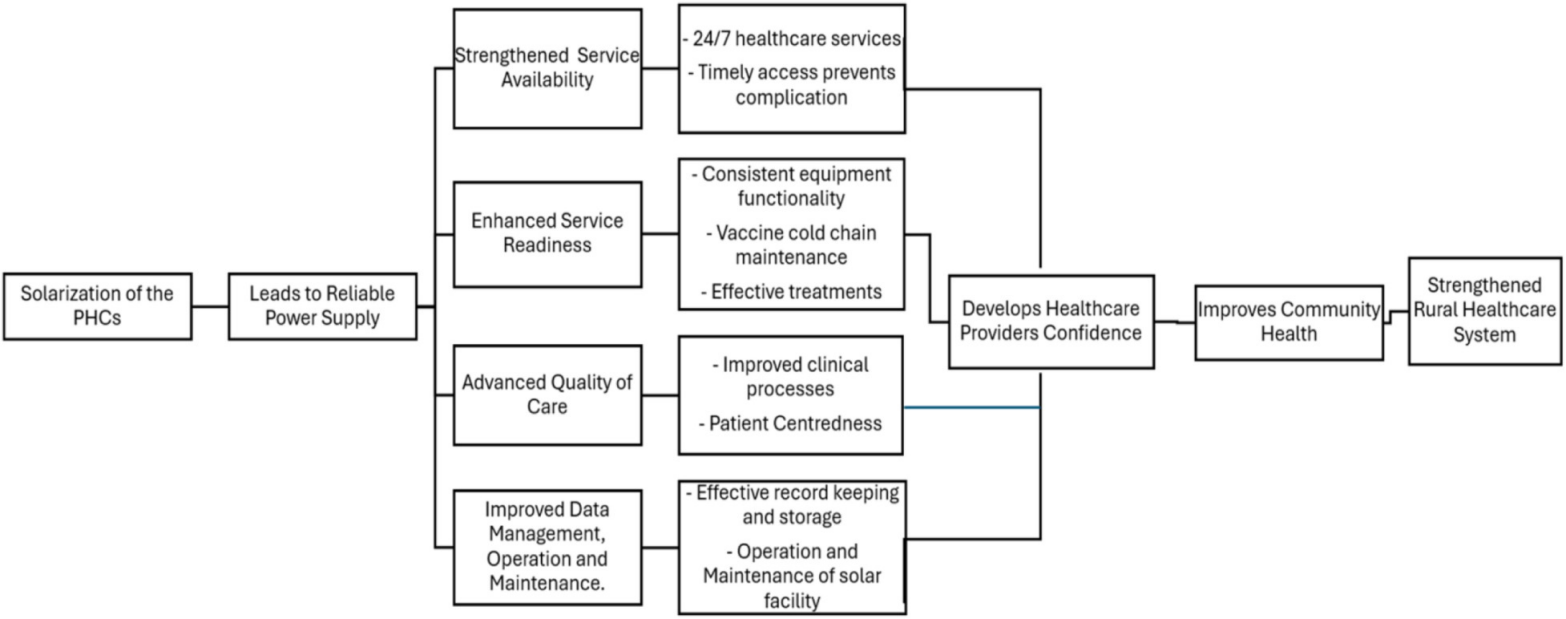
Solarisation of PHCs for strengthened rural healthcare system through HHFA framework.

Figure 1 illustrates the conceptual framework, showing the progressive impact of solarisation at the PHCs by representing the pathways through which reliable power supply catalysed by solar energy strengthens the rural healthcare system. The framework proposed that solarisation of PHCs leads to improved healthcare services, serving as an important determinant for significant improvement. Reliable power supply through solar energy strengthens service availability, enabling round-the-clock healthcare and timely interventions to prevent complications. It also improves service readiness by ensuring consistent equipment functionality, maintenance of the vaccine cold chain, and facilitation of effective treatments. Advanced quality of care is achieved through improved clinical processes and greater patient-centred care, while robust data management practices lead to effective record-keeping, health information systems, and sustainable operation and maintenance of solar infrastructure. These interlinked pathways foster increased confidence among healthcare providers, improving overall community health outcomes. The convergence of these systematic gains strengthens rural healthcare systems, making them more resilient, patient-centred, and capable of meeting the emerging needs of the community.

Together, these four indicators comprehensively capture the capacity of health facilities to deliver quality, accessible, reliable, and well-managed healthcare services, making HHFA a robust conceptual framework for assessing the healthcare facility level impacts with solarisation as a catalyst for improved rural and remote—primary healthcare facilities and its impact on health outcomes.

## 3 Results

### 3.1 Theme: strengthened service availability

Theme 1 emphasised the improved energy reliability that enabled uninterrupted provision of essential health services, including emergency care, diagnostics, and clinical services.

#### 3.1.1 Subtheme: emergency and nighttime provisions

The implementation of solar energy improved emergency response and nighttime healthcare services in rural PHCs. Previously, healthcare providers struggled to manage critical care like sudden accidents, snake bites, scorpion bites, and poisoning incidents using inadequate lighting from the mobile torches and candles. This limitation not only compromised patient safety but also undermined community trust in PHCs. Solar power transformed this scenario by ensuring reliable facilities during emergencies, enabling providers to deliver immediate and appropriate care, regardless of the time or day. This reliability instilled community confidence and provided a sense of security to both patients and staff, transforming the PHC into a true 24/7 health provider during critical incidents. As a result, the PHCs are now viewed as dependable healthcare hubs, even when villages are without electricity.

During night emergencies like snake bites, scorpion bites, and poison consumption cases, solar energy has transformed our ability to provide immediate care. Previously, community members questioned why the PHC existed when we couldn’t even provide basic lighting during emergencies. [Staff Nurse, Female, 48]

For the last 15 years, we struggled with the nighttime deliveries, IPD and OPD services using mobile torches. Solar implementation has enabled us to provide consistent 24-h healthcare services regardless of the grid availability. [Staff Nurse]

Nighttime services have improved drastically. Patients feel safe coming to the PHC for treatment, especially during the rainy season when the power cuts are frequent. They know the PHC has reliable power through solar energy. [Staff Nurse]

#### 1.1.2 Subtheme: service continuity and climate resilience

Rural PHCs face significant challenges during adverse weather conditions such as storms, heavy rains, and high winds, which frequently damage power infrastructure and critical equipment at the PHCs, making healthcare service challenging. Solar energy implementation has created unprecedented service continuity by providing reliable power even when the grid electricity fails for days. This weather resilience ensures that healthcare services remain uninterrupted during critical periods when communities are most vulnerable. The transformation is so significant that villagers now recognise PHCs as reliable power sources during power outages, often coming to charge their phones and seek assurance that healthcare service remains available.

When transformers burned down, solar energy enabled us to continue providing services effectively without interruption. Previously, power outages lasting 3–4 days completely stopped our operation. [Group D]

During heavy winds and rains when trees and poles fall, causing day-long power outages, solar ensures uninterrupted services. Villagers now come to charge their phone here, knowing the PHC runs on solar even when the entire village is without power. [Group D]

### 3.2 Theme: enhanced service readiness

Theme 2 highlighted how reliable power supply enhanced facility readiness by ensuring functional medical equipment, cold chain maintenance, and improved working condition for healthcare staff.

#### 3.2.1 Subtheme: vaccine storage and cold chain management

One of the most critical impacts of solarisation has been the enhancement of vaccine storage and cold chain management. Previously, frequent power cuts lasting 2–3 days would compromise vaccine potency, leading to significant financial losses. Ice packs became watery and vaccines worth lakhs were destroyed, undermining immunisation services. Solar power has revolutionised this scenario by ensuring continuous refrigeration and proper temperature maintenance for vaccines and ice packs.

Vaccine storage has been revolutionizing with continuous cooling and proper ice pack maintenance. Previously, vaccines worth lakhs of rupees could get destroyed due to power cuts, but now we have reliable vaccine storage throughout summer.

Vaccine spoilage has been eliminated. Previously, two-three days power cuts would cause vaccines to lose their potency. Now, with solar energy, our immunization program - one of the main health programs at the PHC operates with fully safe vaccines.” [Health Information Office, Male, 45]

Ice packs used to become watery and vaccine spoilage was common. Post solarisation, the ILR freezer maintains proper temperatures consistently. [ASHA, Female, 49]

#### 3.2.2 Subtheme: equipment and infrastructure readiness

Solar implementation has significantly improved the operational readiness of medical equipment and infrastructure at PHCs. Previously, healthcare providers had to search for alternative backup options or transport vaccines to other facilities during power failure, causing delays and disrupting service delivery. The self-sufficiency achieved through solar power means that PHCs can now manage all equipment operations independently—from vaccine refrigerators to baby warmer and diagnostic equipment.

Previously we had to transport vaccines to other PHCs during power issues. Post solar implementation, we can manage proper vaccine storage to our own facility level without depending on external support. [Staff Nurse. 47]

We no longer search for alternative backup options, even during electricity issues, we can manage at our facility level. Vaccination sessions are now conducted on time without delays caused by transporting vaccine to other PHCs [Staff Nurse, Female, 38]

### 3.3 Theme: advanced quality of care

Theme 3 emphasised how consistent electricity contributed to better quality of care through safer procedures and increased patient confidence in facility-based services.

#### 3.3.1 Subtheme: maternal and child health improvements

Solar energy has drastically improved maternal and child health outcomes by ensuring proper lighting, heating, and cooling during deliveries and postnatal care. The transformation from conducting deliveries using torchlight or candlelight to reliable lighting and fan facilities represents a fundamental improvement in care quality. Baby warmers now operate continuously, instead of providers having to cover newborns with cloth for warmth. Most significantly, patients now extend their hospital stay after delivery, allowing for better recovery and reduced infection risks.

We have delivered babies using torchlight previously. Now with solar implementation, deliveries happen under proper lighting with fan facilities available 24 h significantly improving maternal and child outcomes. [Staff Nurse, 45]

Patients now extend their stay for two additional days post delivery instead of leaving immediately, reducing infection risks. Proper lighting and fan facilities enable comprehensive post delivery care and safe discharge. [Staff Nurse, 39]

Baby warmers now run reliably instead of covering babies with cloth for heat, we are confident that even without grid power, we can manage effectively. It’’s the best thing that has happened to our facility. [Medical Officer, 42]

#### 3.3.2 Subtheme: patient care environment and staff motivation

The improved patient care environment created by reliable solar power has had a cascading effect on both patient satisfaction and staff motivation. Continuous light and fan facilities during extreme health conditions enable healthcare workers to provide care for extended periods without fatigue or discomfort. Patients actively choose to visit PHCs specifically because of the availability of comfortable amenities like fans and proper lighting, which was not the case previously.

Hot water facilities for pregnant women and water filtration for preventing non communicable diseases are now reliably available. During summer heat stress, continuous fan operations enable staff to work efficiently and provide quality care. [AYUSH Medical Officer, Male, 57]

Continuous and reliable lighting and fan facilities have motivated me to work for longer period. Patients now come to the sub-centres as well, specifically because of the fan and lighting facilities available. [Community Health Officer, Female, 37]

### 4.1 Theme: improved data management, costs and, operation and maintenance

Theme 4 demonstrated that electrification strengthened health information management systems while reducing operational costs and improving efficiency in facility operation and maintenance.

#### 4.1.1 Subtheme: cost reduction and financial sustainability

The financial impact of solar implementation has been substantial, with PHCs reporting electricity bill reductions from Rs. 18,000—Rs. 20,000 to as low as a minimum maintenance charge of Rs. 2,000 per month, with savings of 50%–60% in operating expenses. These cost reductions have enabled PHCs to redirect funds towards patient care and facility improvements rather than utility expenses.

Power bills have reduced drastically from Rs. 18,000–Rs. 20,000 to a minimum charge of Rs. 2000. Solar implementation has provided power connection to the entire healthcare instead of limited specific rooms like before. [ASHA, Female, 47]

The hospital runs completely on solar with a 50%–60% reduction in power bills, our staff has received the O and M training, ensuring sustainable management of the solar system. [Medical Officer, Male, 45]

#### 4.1.2 Subtheme: digital health management and administrative efficiency

Reliable power supply through solar energy has improved digital health information systems and administrative processes at the PHCs. Healthcare providers can now maintain laptops and computer systems continuously, enabling real-time data entry and regular updates to health information systems. The ability to generate reports and maintain digital records without interruption has streamlined healthcare delivery processes, reducing patient treatment times.

Laptops and computer systems enable us to keep health data updated on a daily basis with reliable energy support. Healthcare information data can be properly maintained and uploaded weekly without interruptions. [Group D, Male, 39]

Solar energy has been helpful for printouts and maintaining health information data uploads. Patient treatment time has reduced by half hour from previously one hour due to improved operational efficiency. [Group D, Male, 35]

#### 4.1.3 Subtheme: sustainable operation and maintenance

The sustainability of solar systems at PHCs is ensured through proper training and responsible usage practices. Healthcare staff have received training on operation and maintenance, enabling them to manage the system effectively and troubleshoot minor issues independently. Providers also demonstrate environmental consciousness by using lights and fans judiciously, only when needed.

We maintain the solar energy system responsibly, using lights and fans only when needed. Staff training on solar maintenance and operation should be provided continuously to all team members with clear responsibilities. [Pharmacist, Male, 45]

## 4 Discussions

This study explored the impact of solarisation of PHCs from a healthcare providers’ perspective in Yadgir, an aspirational district of Karnataka. Drawing on the HHFA framework and field-level insights from FGDs and IDIs, findings revealed improvements across all four domains due to solarisation of the PHCs. Service availability is the first domain, highlighting the role of solar energy in making services available at any hour of the day for any emergency, with nighttime provisions, also ensuring continued service during external climatic-induced situations. Theme two focuses on service readiness enabled by realible electricity, ensuring proper vaccine cold chain maintenance and fucntioning of essential medical equipment such as baby warmer, oxygen concentrator and other critical devices required during healthcare emergencies. The third domain is advanced quality of care, highlighting how solarisation improved maternal and child healthcare, enhanced patient-centred care environments, and increased staff motivation. Theme four highlights improved healthcare data management, efficient operations and maintenance of the solar energy and it has reduced electrcity cost by nearly 50-60% allowing the savings to be used towards health facility improvements. Overall, solarisation of PHCs improved healthcare services for communities while enhancing healthcare provider safety, motivation, and satisfaction in serving rural and remote communities. Healthcare providers consistently emphasised the importance of reliable energy supply as a foundation for effective treatment of pregnant women, newborns, and individuals requiring immediate care, thereby bridging gaps in rural health provision. These findings align with previous studies demonstrating that reliable electricity significantly improves healthcare service delivery. For example, improved lighting has been associated with a 76% increase in healthcare worker satisfaction and improved emergency response capacity (15). Similarly, solar electrification interventions have been shown to increase 24-h emergency service availability from 7.7% to 100% in electrified sites (16). The “We Care Solar” initiative in Zimbabwe enabled more than 1,80,000 safe deliveries annually, reducing the risk of infections and improving post-delivery monitoring (17). Studies from Indian PHCs report 40%–50% reductions in monthly electricity bills and 90% operational cost savings compared to diesel generators (18). Healthcare resilience highlights the importance of robust infrastrucutre in maintaining service continuity during emergencies. Reliable energy supply through solar enregy stregthens the ability of PHCs to minimize service disruptions and sustain continous healthcare services (19). Increased access to regular and reliable power supply at PHCs is linked with higher health workforce availability and greater utilisation of core services such as vaccination and deliveries (20). Electrification of health facilities is further associated with improvement in health worker motivation, retention, and perceived quality of care (21). Reliable electrification through solar energy improves operational efficiency and reduces dependency on informal coping mechanisms such as service referrals and delayed care (22). Energy access strengthens health system performance by enabling the continuity, safety, and efficiency of clinical services at PHCs (23). Health system frameworks increasingly recognise solar energy as a foundational input alongside workforce, medicines, and governance (24).

Operation and maintenance remain critical aspects following implementation of solarisation. Routine maintenance is essential to ensure smooth functioning of PHC services during emergencies. The routine maintenance of the solar panel and batteries emerged as one of the critical concerns highlighted by the healthcare providers. They emphasized that regular maintenance of these panels are essential for ensuring system efficiency and longevity. To ensure this, their staffs receive Operation and Maintenance (O&M) training on regularly basis. Additionally, a Remote Monitoring System (RMS) is used to track system performance for timely detection of defects and fixing these issues efficiently.

### 4.1 Limitations

Despite the positive impacts reported, several limitations remain. Healthcare providers highlighted challenges related to system maintenance, unclear protocols for repair and component replacement, and limited local technical troubleshooting capacity, compromising long-term sustainability of solar systems at PHCs. Addressing these issues requires clearly defined maintenance frameworks, technical support mechanisms, and regular training programmes for facility staff. Future research should examine the cost-effectiveness and long-term sustainability of solar electrification in rural healthcare facilities, including initial capital investment, operational costs, and long-term maintenance requirements.

## 5 Conclusions

The collective insights from healthcare providers highlight the transformative role of solarisation in improving healthcare delivery by ensuring reliable energy access and strengthening facility performance. Building upon the WHO HHFA framework, the findings reveal that solarisation has substantially enhanced service availability and readiness, advanced quality of care, improved data management systems, reduced costs, and improved operational efficiency and maintenance. The availability of uninterrupted power ensures effective functioning of life-saving equipment, maintenance of the vaccine cold chain, and timely emergency response—all of which are integral to the overall quality and reliability of healthcare services. The healthcare providers emphasized that successful solarization of PHCs goes beyond mere installation. For effective functioning of solarization, it must be supported by the regular operations and maintenance, timely technical support for repairs and troubleshooting, and smooth integration into the routine health system operations ensuring reliability. When these enabling conditions are effectively in place, solar energy becomes a powerful catalyst not only for improving healthcare quality but also for enhancing provider satisfaction and community trust. Ultimately, the solarisation of PHCs contributes significantly towards achieving UHC by fostering resilient, reliable, and sustainable healthcare systems in rural and remote regions of India.

## 6 Policy implications

This study examined the impact of solarisation of PHCs from healthcare providers’ perspectives, focusing on service availability, readiness, improved quality of care, and health data management through the HHFA framework. The findings hold critical implications for policies aimed at strengthening energy reliability and health system performance in rural India. Solarisation emerged as a sustainable strategy for improving healthcare outcomes, particularly in the most backward and poor districts. However, qualitative insights revealed continuing challenges in terms of the limited technical support and inadequate maintenance of solar energy systems at PHCs, reducing its long term effectiveness, sustainability and reliability. Therefore, the following actions are required:

- System level: allocate dedicated budgets for energy maintenance.
- Facility level: train the healthcare staff to manage the basic operations and maintenance of solar system, with simplified processes enabling them to report any faults or disruptions.
- Community level: involve local governance bodies in maintaining the solar system.

Strengthening the energy–health policy interface is essential for achieving universal healthcare coverage for all in rural and remote areas.

## Data Availability

The original contributions presented in this study are included in the article/Supplementary Material, further inquiries can be directed to the corresponding author/s.
